# Abatacept for severe anti-TNF-alfa refractory JIA-associated uveitis: one year follow-up

**DOI:** 10.1186/1546-0096-9-S1-P189

**Published:** 2011-09-14

**Authors:** MG Alpigiani, P Salvati, S Callegari, A Bagnis, M Papadia, CE Traverso, R Lorini

**Affiliations:** 1 Department of Pediatry, Gaslini Institute, Genoa, Italy; 2 Eye Clinic, Department of Neurosciences, Ophthalmology and Genetics, University of Genoa, Genoa, Italy

## Introduction

Uveitis in JIA can be severe and immunosoppressive therapies may not be sufficient. Anti-TNF-alfa agents (Infliximab, Adalimumab) have been proposed. The percentage of success is different among series and no controlled trials have been published yet. Abatacept, a selective T-cell co-stimulation modulator, has been shown to be a valid alternative to anti-TNF-alfa agents in patients with refractory uveitis (Angels-Han S.2008; Zulian 2010).

## Case report

We report on a 20-years-old girl, affected since one year of age, by a severe form of poliarticular JIA. She received immunosuppressors (Metrotrexate, Azathioprine…) associated with steroids, with no articular benefit. When Enbrel plus Metrotrexate was started, she got into remission. After one year she presented uveitis bilaterally, so steroids were started again. She obtained partial ocular improvement, receiving Infliximab plus Metrotrexate, but the former was suspended because of an adverse reaction (dyspnea, rash) and Adalimumab was started, with no side effects. After three years, she had flare for uveitis and arthritis. Adalimumab was suspended and Abatacept was started, with no side effects. After 12 months, uveitis seems to be stable, while arthritis, after a good initial response, seems to be active again.

## Conclusions

In our case, Abatacept was a valid drug in uveitis treatment, less effective in arthritis. In the literature, there are other reports of good response of ocular inflammation, with reduced effectiveness on articular flares (Kenawy N., 2011). Long- term follow-up in patients with severe uveitis and JIA treated with Abatacept is necessary to check if this drug is a valid alternative to anti- TNF drugs.

